# DNA metabarcoding of benthic algae and associated eukaryotes from Lake Baikal in the face of rapid environmental changes

**DOI:** 10.18699/VJGB-22-12

**Published:** 2022-02

**Authors:** Yu.S. Bukin, L.S. Kravtsova, T.E. Peretolchina, A.P. Fedotov, A.E. Tupikin, M.R. Kabilov, D.Yu. Sherbakov, E.V. Mincheva

**Affiliations:** Limnological Institute of the Siberian Branch of the Russian Academy of Sciences, Irkutsk, Russia; Irkutsk State University, Irkutsk, Russia; Limnological Institute of the Siberian Branch of the Russian Academy of Sciences, Irkutsk, Russia; Limnological Institute of the Siberian Branch of the Russian Academy of Sciences, Irkutsk, Russia; Limnological Institute of the Siberian Branch of the Russian Academy of Sciences, Irkutsk, Russia; Institute of Chemical Biology and Fundamental Medicine of the Siberian Branch of the Russian Academy of Sciences, Novosibirsk, Russia; Institute of Chemical Biology and Fundamental Medicine of the Siberian Branch of the Russian Academy of Sciences, Novosibirsk, Russia; Limnological Institute of the Siberian Branch of the Russian Academy of Sciences, Irkutsk, Russia; Novosibirsk State University, Novosibirsk, Russia; Limnological Institute of the Siberian Branch of the Russian Academy of Sciences, Irkutsk, Russia

**Keywords:** algal communities, metabarcoding, 18S rDNA, Illumina MiSeq, Lake Baikal, green algae, Spirogyra, водорослевые сообщества, метабаркодинг, 18S рРНК, Illumina MiSeq, озеро Байкал, зеленые водоросли, Spirogyra

## Abstract

Here we report new data describing the biodiversity of phytobenthic communities based on DNA-metabarcoding using the 18S rDNA marker and the Illumina MiSeq system. The study was initiated due to the blooming of f ilamentous algae (mainly of the genus Spirogyra) and cyanobacteria in the coastal zone of Lake Baikal under climate change and anthropogenic impact. The composition and taxonomic diversity of algae and other organisms associated with them on different sites of Lake Baikal (near Bolshoi Ushkaniy Island, in Listvennichny Bay) and in the Kaya (within the city of Irkutsk, located in the same drainage basin as Lake Baikal) were determined using DNAmetabarcoding. About 15 thousand reads of the 18S rRNA marker were obtained by applying NGS (next-generation sequencing). The species of algae dominating in the number of reads, as well as the diff icult-to-identify taxa (Stramenopiles, Alveolata, Euglenozoa, Chromista, Rhizaria, Amoebozoa, etc.), which play an important role in the functioning and formation of the structure of algal communities, were revealed. The Shannon index of the communities studied ranges from 1.56 to 2.72. The advantages and weaknesses of using DNA-metabarcoding based on the 18S rRNA gene fragment for studying the structure of algal communities are shown. The advantage of this method is the possibility to more fully determine the diversity of eukaryotes taxa, which are diff icult to identify by morphology, without involving a large number of specialists, while the disadvantage of the method is the distortion that may occur during the PCR. Here, ways of solving this problem are proposed. The results of the study show that the analysis of the minor component of the eukaryotic community in samples (organisms with low biomass) consisting of a mixture of multicellular and unicellular organisms requires a read-depths of at least 100,000 sequences per sample. In general, the DNA-metabarcoding method is recommended for studying the structure of algal communities and eukaryotes associated with them.

## Abstract (ru)

Впервые приводится оценка разнообразия фитобентосных сообществ на основе ДНК-метабаркодинга с использованием ампликонов фрагмента гена 18S рРНК и технологии Illumina MiSeq. Исследование проведено в связи с цветением нитчатых водорослей (преимущественно рода Spirogyra) и цианобактерий в прибрежной зоне озера Байкал в условиях изменения климата и антропогенного воздействия. С помощью ДНК-метабаркодинга определен видовой состав водорослей, а также таксономическое разнообразие ассоциированных с ними эукариот в разных районах Байкала (у острова Большой Ушканий, в заливе Лиственничный) и в р. Кая (в черте г. Иркутска), находящейся в одном водосборном бассейне с оз. Байкал. С помощью NGS (next generation sequencing) получено более 15 тыс. прочтений 18S рРНК маркера. Выявлены виды водорослей, доминирующие по количеству прочтений, а также трудно идентифицируемые таксоны Stramenopiles, Alveolata, Euglenozoa, Chromista, Rhizaria, Amoebozoa и др., играющие важную роль в функционировании и формировании структуры водорослевых сообществ. Охарактеризовано разнообразие грибов и грибоподобных организмов в изучаемых сообществах. Индекс Шеннона рассмотренных сообществ колеблется от 1.56 до 2.72. Показаны преимущества и слабые стороны использования ДНК-метабаркодинга на основе фрагмента гена 18S рРНК для изучения структуры сообществ водорослей. Метод позволяет более полно учесть разнообразие таксонов эукариот, трудно идентифицируемых по морфологии, без привлечения большого числа специалистов, что характеризует его преимущество. Недостатком метода являются искажения, возникающие при проведении ПЦР. Предложены пути решения для устранения этого недостатка. Результаты исследования показывают, что для анализа минорной компоненты сообщества эукариот в образцах (организмы с малой биомассой), состоящих из смеси многоклеточных и одноклеточных организмов, требуется глубина прочтения не менее чем 100 000 последовательностей на пробу. В целом метод ДНК-метабаркодинга рекомендован для исследования структуры сообществ водорослей и ассоциированных с ними эукариот.

## Introduction

Recently, a number of catastrophic and rapidly developing ecological phenomena, including the expansion of filamentous Chlorophyta and Cyanobacteria, took place in some areas of the coastal zone of Lake Baikal (Timoshkin et al., 2016). The first reports of such changes appeared in 2011 (Kravtsova et al., 2012, 2014). Previously, no changes in the coastal zone of the lake had been observed, with the invasion of Elodea canadensis in the 1970s being the only exception (Izhboldina, 1990).

It is known that the algae of Lake Baikal are characterized by zoning in spatial distribution and seasonal dynamics, which persisted for a long time (Meyer, 1930; Izhboldina, 1990, 2007; Izhboldina et al., 2017). However, since 2011, researchers have begun to note the overgrowth of the bottom with filamentous algae in Listvennichny Bay (Kravtsova et al., 2012, 2014). The overgrowth of the bottom with filamentous algae is also recorded in other areas of the lake near the settlements Kultuk, Baikalsk, Severobaikalsk (Timoshkin et al., 2018; Kravtsova et al., 2020). Among the filamentous algae blooms in the littoral zone near the Listvyanka, representatives of the genus Spirogyra dominate. For the first time (in almost 100 years of research), benthic filamentous algae Spirogyra were found in the plankton communities of the coastal zone (Bondarenko, Logacheva, 2016). In some areas of Lake Baikal, large accumulations of algae washed up on the shore were recorded (Suturin et al., 2016; Timoshkin et al., 2016, 2018). Forming algal mats along the coastline, filamentous algae impede the penetration of light, concentrate suspension and thus negatively affect filter feeders organisms; in particular, Baikal sponges (Khanaev et al., 2018). It should be noted that algae of the genus Spirogyra were encountered in Lake Baikal earlier. Researchers have occasionally found single spirogyra filaments in bottom phytocenoses in the bays of Lake Baikal. Among them, 4 species of the genus Spirogyra and 3 forms were registered: S. calospora, S. decimina (S. decimina f. jurgensis, S. decimina f. longata), S. weberi (S. weberi f. weberi), S. hassallii (Izhboldina, 2007). Later, the species S. f luviatilis was discovered. This species dominated in the littoral zone of Listvennichny Bay in accumulations of filamentous algae in 2012 (Timoshkin et al., 2014). It is possible that endemic species of the genus Spirogyra, which have adapted to the specific conditions of the lake’s ecosystem, can also inhabit Baikal. Currently, the question about the number of species occurred in accumulations of filamentous algae remains open.

A change in the composition of algae communities (and the ratio of algae biomass) entails a change in the composition of eukaryotic organisms associated with them (unicellular algae, protozoa and fungus-like organisms). The role of parasitic forms of eukaryotes (fungus-like organisms), negatively affecting the development of algae typical of the littoral zone, is practically not studied.

Algae of the littoral zone of open Lake Baikal are a rather complex object for taxonomic identification. Therefore, the analysis of the species diversity of algae and their abundance in benthic communities using classical morphological and hydrobiological methods is a laborious and time-consuming process. An even more difficult task is to study the taxonomic composition and quantitative ratio of various groups of eukaryotic organisms (including microeukaryotes) associated with algae. Identification of such taxa may require long-term cultivation on selective media and laborious microscopic analysis

The application of modern molecular genetic approaches, such as DNA metabarcoding, can simplify and speed up these studies. This method includes the amplification of universal genetic markers in a mixture of DNA extracted from a sample followed by next generation sequencing (NGS) and analysis of the obtained sequence data set. Metabarcoding allows the detection of all species present in the bulk of DNA extracts and determines the species composition and quantitative ratio of taxa.

The metagenomic analysis of amplicons was widely used in the study of bacterial community composition using the

universal marker 16S ribosomal DNA (Petrosino et al., 2009). Similar studies were carried out for different bacterial communities of Lake Baikal (Kurilkina et al., 2016). The number of studies where metabarcoding based on 18S rDNA fragments and Folmer fragment of COI of mtDNA is used for analysis of eukaryotic communities is continuously increasing (Leray et al., 2013; Taylor, Cunliffe, 2014; Hawkins et al., 2015; Smith et al., 2017). These markers were implemented for studying communities from Lake Baikal. In particular, 18S rDNA was used for metabarcoding micro-eukaryotic communitie (Yi et al., 2017), and COI was used for studying invertebrates’ communities (Metazoa) (Kravtsova et al., 2021). Data on the DNA-metabarcoding of benthic algae in Lake Baikal are extremely limited (Mincheva et al., 2017), and data on algae communities and eukaryotes associated with them are currently lacking.

18S rRNA is most suitable for studying algal communities and associated organisms. To amplify various regions of this gene, universal primers that cover a wide range of species belonging to different distant taxa have been developed. Interpretation of sequencing results is facilitated by the availability of databases containing templates that allow alignment of large arrays of sequences, taking into account the secondary structure.

The aim of the study was to test the DNA-metabarcoding approach using the 18S rRNA marker to assess the diversity of benthic algal communities and associated eukaryotic organisms.

## Materials and methods

The sampling of algae (meio-, macrophytes ≥2 mm in size) was carried out in July–August 2015 on a stony littoral near Bolshoi Ushkaniy Island in Northern Baikal (background area, stony littoral), Listvennichny Bay opposite Listvyanka village in South Baikal (area overgrowing of the bottom with filamentous algae, stony littoral). For comparison, sample was collected in the Kaya River, flowing within the city of Irkutsk, located in the same drainage basin as Lake Baikal (Table 1).

**Table 1.**
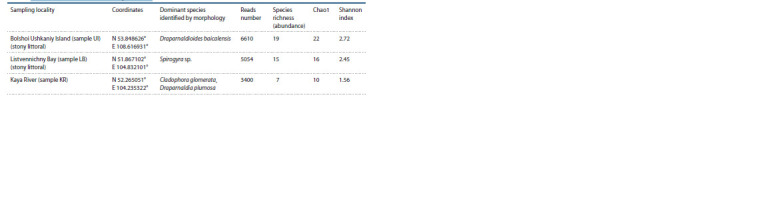
General characteristics of the sample studied

In Northern and Southern Baikal, algae were collected by divers from three depths: 0–2, 2–5 and 6–10 m, and in the Kaya River – from a depth of 0.05–0.10 m. In each sampling site, algae collected from different depths were combined into one integral sample. The identification of algae was carried out according to L.A. Izhboldina (2007).

For molecular genetic analysis, the collected algae samples were fixed with 80 % ethyl ethanol, and then refixed with 70 % ethanol a day later

Total DNA was isolated according to the modified method of Doyle and Dickson (Doyle, Dickson, 1987). A fragment of the 18S rRNA gene was used as a molecular genetic marker (Katana et al., 2001). Amplification was carried out with a set of PCR reagents with HS-Taq (Biolabmix, Novosibirsk, www.biolabmix.ru) in 25 μL of the reaction mixture in a Bio-Rad-T100 thermal cycler (Bio-Rad, USA). The genetic marker (about 400 base pairs in length) encoding the V1–V2 variable region of 18S rRNA was amplified using the 18SF universal primers: 5′-AACCTGGTTGATC CTGCCAGT-3′ and 416-37R: 5′-ATTTGCGCGCCTGCT GCCTTCC-3′ (Katana et al., 2001). The amplification conditions were as follows: predenaturation at 95 °C for 5 minutes, then 25 cycles: denaturation at 95 °C for 1 minute, annealing of primers at 55 °C for 1 minute, elongation at 72 °C for 2 minutes (5 minutes on the last cycle)

The reaction products were analyzed by electrophoresis in 1 % agarose gel. The band of the expected size was excised and purified using an agarose gel DNA elution kit (Biosilica, Novosibirsk).

The paired DNA sequencing of amplification products was performed using the Illumina MiSeq technology at the SB RAS Genomics Core Facility of the Institute of Chemical Biology and Fundamental Medicine, Siberian Branch of the Russian Academy of Sciences (Novosibirsk, Russia).

All stages of the analysis of Illumina MiSeq DNA reads were carried out using the MOTHUR program (Schloss et al., 2009) and the SILVA 18S rRNA sequence database (Quast et al., 2012) according to the MiSeq standard operating procedure (MiSeq SOP) (Kozich et al., 2013). The analysis consisted of the following procedures: (1) merging of paired MiSeq reads of amplification products into consensus sequences; (2) trimming of cosensus sequences by reading quality (deleting sequences with an average quality below 20 units); (3) removal of chimeric sequences from the data set; (4) deletion of sequences that do not correspond to the amplified 18S rRNA fragment in the SILVA database; (5) aligning sequences according to the SILVA database template; (6) calculation of the matrix of genetic distances (the proportion of mismatched nucleotides in pairwise comparison of sequences was used as a metric of distances); (7) clustering of sequences based on genetic distances; (8) identification of OTUs (operational taxonomic units) at the level of cluster distance (0.01) corresponding to interspecific differences (1 %); (9) drawing up of a table indicating the number of sequences per OTU in the sample; (10) secretion of representative sequences for each OTU; (11) taxonomic identification of representative sequences using the online BLAST application

The statistical convergence of the results of assessing taxonomic diversity was characterized using saturation curves and the Chao1 index (Chao, 1987). The Chao1 index gives an estimate of the expected α diversity in the studied community based on the observed number of taxa at the current number of reads per sample. In other words, Chao1’s calculations allow the researcher to understand how many more taxa (species) can potentially be found in a sample if the number of reads increases from the existing value to infinity. A significant excess of the expected α diversity calculated using the Chao1 index over the observed one indicates an insufficient number of reads in the sample and a loss of taxa.

Data on the taxonomic composition of communities (representation of OTU species rank) were compared using cluster analysis by the UPGMA method, where the Bray–Curtis dissimilarity coefficient was used as a measure of distance. Before clustering, the data on the number of sequences per each presented OTU in the samples were normalized to the average number of reads per sample. The structure of the identified communities was visualized on a heat map.

The diversity of communities was assessed by the Shannon index, as well as by the species rank abundance curve, taking into account that the slower the abundance curve goes to zero, the more diverse the community is.

Statistical calculations (diversity index, clustering, data visualization) were carried out using the vegan package (Dixon, 2003) for the R programming language.

## Results

After applying initial data filtering, the dataset included 6610 reads from the Bolshoi Ushkaniy Island, 5054 reads from the Listvennichny Bay and 3400 reads from the Kaya River (see Table 1).

OTUs grouped at genetic distances of 1 % (0.01) had different numbers of sequences: 88 OTUs contained more than one sequence (2 – 4000), and 378 OTUs were presented by a single sequence. The OTUs presented by a single sequence accounted for 2.57 % of the entire dataset, which was lower than the permissible 5 % threshold and indicated the absence of errors in the amplification, sequencing and initial data filtering steps. According to the practice of metabarcoding research (Kozich et al., 2013), only those OTUs that included 4 or more sequences were used for analysis (Table 2).

**Table 2.**
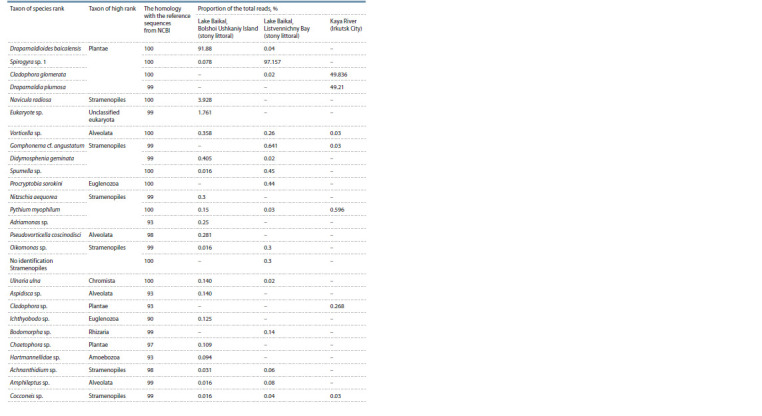
Results of the taxonomic identif ication of OTU based on homology with sequences from the NCBI database

**Fig. 1.**
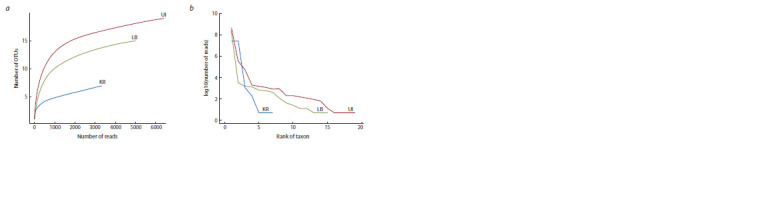
Curves of saturation of the number of taxa of species rank in samples with various sample sizes of reads (a) and curves of the species abundance (b). Here and in Fig. 2 and 3: UI – Lake Baikal, stony littoral, Bolshoi Ushkaniy Island; LB – Lake Baikal, stony littoral, Listvennichny Bay; KR – Irkutsk, Kaya River.

The convergence curves of species abundance in samples from different localities showed the absence of saturation (Fig. 1, a). The same result is provided by the values of the Chao1 index (see Table 1), according to which the number of species in the communities was also underestimated. Moreover, the most underestimated was the species composition of the minor component represented by unicellular eukaryotic organisms

**Fig. 2.**
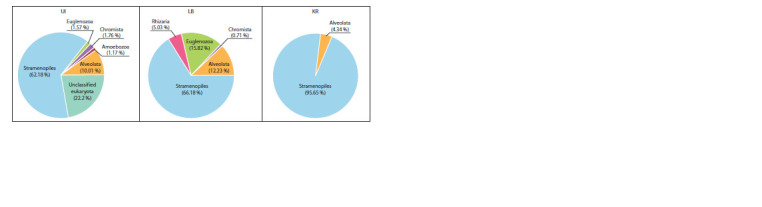
Distribution of high rank taxa associated with algae (percentage ratios from their number of reads).

**Fig. 3.**
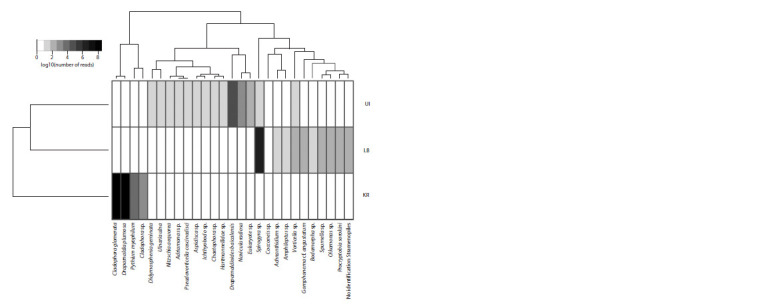
Distribution of high rank taxa associated with algae (percentage ratios from their number of reads). Gray gradient shows the normalized number of reads (in a logarithmic scale) per taxon.

We identified 27 OTUs characterizing different taxa of algae and associated organisms (Fig. 2 and 3, see Table 2). Most taxa identified by BLAST belonged to the Charophyta and Chlorophyta algae: Spirogyra, Draparnaldioides, Cladophora, and Draparnaldia (see Table 2). Dominant taxa identified by DNA-metabarcoding are fully corroborated with those revealed by the morphologic analysis of samples (see Table 1). In addition, a significant proportion of the sequences belonged to high-level taxa: Stramenopiles, Alveolata, Euglenozoa, Chromista, Rhizaria, and Amoebozoa (see Fig. 2). All samples differed by spectrum of dominant species (see Fig. 3, Table 2). Endemic Draparnaldioides baicalensis dominated in the background area near Bolshoi Ushkaniy Island of Northern Baikal. Spirogyra sp. dominated in Listvennichny Bay. Communities of Bolshoi Ushkaniy Island and Listvennichny Bay include 10 common taxa in their composition and form one cluster on the dendrogram (see Fig. 3). Cladophora glomerata and Draparnaldia plumosa dominated in the Kaya River. The community from the Kaya River differed significantly from two other communities in the taxonomic composition and shared two taxa with the community of Bolshoi Ushkaniy Island, and four taxa with the community from Listvennichny Bay.

In general, according to the Shannon index, the communities from the Bolshoi Ushkaniy Island and Listvennichny Bay are more diverse compared to Kaya River (see Table 1). The abundance curves also confirm this result (see Fig. 1, b).

## Discussion

In the coastal zone near the Bolshoi Ushkaniy Island, located in the central conservation area of Lake Baikal, where there is practically no anthropogenic impact, typical representatives of the algal flora of the stony littoral of the lake are endemic species Draparnaldioides baicalensis, D. arnoldii, D. arenaria and Cladophora f loccosa f. f loccosa (Izhboldina, 1990). According to the results of the study, no structural changes were observed in the community near Bolshoi Ushkaniy Island (see Table 2, Fig. 3), although it contained trace amounts of Spirogyra sp. 1. The findings of Spirogyra in this area are due to the circulation currents that exist in Lake Baikal (Kravtsova et al., 2020).

During the summer period, in the littoral zone of Listvennichny Bay (before 2000s), at a depth of more than 1.5 metres, Dermatochrysis reticulata, Didymosphenia geminata, and Nitella flexilis species dominated. However, currently, they have lost the leading role in phytocenoses due to the expansion of green filamentous algae Spirogyra atypical for the open littoral zone of Lake Baikal (Kravtsova et al., 2014, 2020), which is confirmed by our results (see Table 2, Fig. 3). Sequences of 18S rDNA fragment obtained for filamentous algae Spirogyra from Listvennichny Bay were 100 % identical to a previously published sequence Spirogyra sp. 1 found in the littoral of this bay in 2013 (Romanova et al., 2013). Thus, we can assume that during the period from 2011 to 2015, the same species, Spirogyra sp. 1, developed in Listvennichny Bay. The spread of filamentous algae creates stressful conditions for the habitation of algal communities. It is known that there is a decrease in diversity according to Shannon index due to the expansion of species in ecosystems (Ling, 2008; Powell et al., 2013). The same regularities are observed in Listvennichny Bay, where algae typical for this period are suppressed; moreover, the Shannon index is lower here than in the community near Bolshoi Ushkaniy Island (see Table 1). Even less diversity by Shannon index was observed in the Kaya River (see Table 1). But we cannot unambiguously conclude that this community is in stressful conditions, because in the literature, we did not find information on the value of the Shannon diversity index characteristic of communities of bottom algae and associated eukaryotic organisms for river ecosystems. It is possible that the value of the Shannon index obtained for Kaya River is, in principle, typical for such an ecosystem

The composition of eukaryotes associated with algae is quite diverse: Stramenopiles, Alveolata, Euglenozoa, Chromista, Rhizaria, and Amoebozoa. Of particular interest are oomycetes of the genus Pythium found in samples from the littoral zone of Lake Baikal (see Fig. 3, Table 2). It should be noted that at present the mycofauna of Lake Baikal has not been practically studied, although the DNA of representatives of the genus Pythium was found during the study of microeukaryotes of the surface layer of bottom sediments of Lake Baikal (Yi et al., 2017). However, it is known that most of freshwater fungi of the genus Pythium are parasites of green algae (Raghukumar, 1987; Li et al., 2010; Carney, Lane, 2014). An increase in the concentration of Pythium in phytocenoses occurs at the end of the growing season, as it contributes to a more rapid destruction of the primary organic matter of plant origin. It is possible that the algae in the community near Bolshoi Ushkaniy Island ended their growing season, and therefore, they were more affected by parasitic fungi, as evidenced by the greater amount of DNA of P. myophilum in samples from this area compared to samples from Listvennichny Bay. On the one hand, P. myophilum, apparently, has not yet had time to spread in the community of the littoral zone of Listvennichny Bay, since during the study Spirogyra sp. 1 was in a good phenological state. On the other hand, Spirogyra sp. 1 can potentially be resistant to the P. myophilum, in contrast to other typical species (for example, Ulothrix), which are widespread in the littoral of Lake Baikal. Then, in this situation, Spirogyra sp. 1 gains a competitive advantage over other species and becomes a community-forming taxon. The distribution of P. myophilum probably makes a certain contribution to changes in the structure of benthic communities of algae in the littoral of Lake Baikal.

Despite the originality of the results obtained, we would like to draw attention to the features of the use of the DNAmetabarcoding approach in the study of algal communities and associated eukaryotes. Analyzing the 18S rRNA metabarcoding data, we faced the problem of determining the threshold of the genetic distance between species within a genus. For 16S rRNA (a marker for bacterial communities), this distance was chosen as 3 % (0.03) of mismatched nucleotides between the compared sequences (Petrosino et al., 2009; Kurilkina et al., 2016). Some researchers use the same distance to separate the OTUs of the species level for analysis of eukaryotic communities based on 18S rRNA (Yi et al., 2017). If this is justified for microeukaryotes to some extent (due to the high rate of evolution and rapid change of generations), then for multicellular organisms such a measure cannot be used to separate species, since it is proved that 18S rRNA is one of the slowly evolving markers for them (Anne, 2006). Our analysis of the literature data showed that for multicellular algae, the distance threshold corresponding to 3 % of mismatched nucleotides separates not species, but different genera and families (Chen et al., 2012; Romanova et al., 2013; Sherwood et al., 2014; Taylor, Cunliffe, 2014). Thus, if we used a threshold of 3 % for isolating OTUs, then we could not identify species, and the study of species diversity in this group of organisms would become impossible. A more detailed analysis of the published information (Chen et al., 2012; Romanova et al., 2013; Sherwood et al., 2014; Taylor, Cunliffe, 2014) allowed us to choose a threshold of 1 % (0.01) substitutions for separating different species within one genus and use it in our study.

In the course of the study, it was found that with 3400– 6610 reads of the 18S rRNA fragment for each sample, the species diversity of eukaryotic organisms associated with benthic algae is clearly underestimated. In the DNA mixture consisting of macro and microorganisms, the main pool of reads was presented by multicellular species (algae), and accounted for from 91 to 99 % of all 18S rRNA reads in each sample. In this case, we adequately evaluate the species diversity of macroorganisms (algae), but lose most of the diversity of eukaryotic organisms associated with them. In the methodological works on the study of bacterial communities based on 16S rRNA metabarcoding (Bukin et al., 2019), it is given that the final number of filtered in quality reads in the sample for an adequate assessment of the species diversity of microorganisms should be 10,000. Considering this, in order to evaluate the species diversity of the minor component of the community of eukaryotic organisms associated with algae, it is necessary to increase the number of reads per sample 50 or more times. Then the minor component will account for approximately 10,000 DNA sequences. In total, several hundred thousand reads will be required. With the current level of development of NGS technologies, this is a completely accessible task. Another way to obtain 10,000 readings on the minor component of the community is a preliminary mechanical separation of the sample into two parts: one of them will include algae, and another will contain organisms associated with algae. In this case, the DNA of the two selected parts should be sequenced separately.

It should also be noticed that the specificity of the universal primers varies for different taxa in samples, and a distortion of DNA-metabarcoding results may arise. As a result, the initial DNA concentration of different taxa in the sample is changed after PCR. It is possible to decrease this effect by reducing the number of PCR cycles during the preparation of a sample for sequencing, using the same sets of reagents and standardizing the sampling method. Another important note refers to the methodology of statistical analysis, which must contain the data range stage. To do this, you can convert the number of reads to the proportion of reads on a taxon in a sample, or normalize the entire dataset (as done in our study) to the average number of reads on the sample. Such data rationing, despite the distortions associated with PCR, will allow determining the trend of changes in the concentration of DNA of any taxon in different compared natural samples using multidimensional statistics (clustering methods, etc.).

## Conclusion

Due to the growth of filamentous green algae, one of the Spirogyra species began to dominate in the community of the littoral in Listvennichny Bay. DNA of this species was also found in the samples of the background area near Bolshoi Ushkaniy Island of Lake Baikal. DNA-metabarcoding based on a fragment of the 18S rRNA gene is a perspective method for studying the structure of algae communities and makes it possible to obtain statistically representative results. This method is also effective for accurate taxonomic identification of a morphologically complex group of organisms, such as filamentous algae. At the same time, DNA-metabarcoding allows determining the representation in the samples of difficult-to-study taxa associated with algae, which play an important role in the formation of the diversity and the functioning of the communities. For a representative assessment of the minor component of the community (eukaryotic organisms associated with algae), a significant increase in the sample sizes of DNA sequences is necessary.

## Conflict of interest

The authors declare no conflict of interest.
